# The paternally imprinted *DLK1-GTL2* locus is differentially methylated in embryonal and alveolar rhabdomyosarcomas

**DOI:** 10.3892/ijo.2013.2153

**Published:** 2013-10-29

**Authors:** GABRIELA SCHNEIDER, MARK J. BOWSER, DONG-MYUNG SHIN, FREDERIC G. BARR, MARIUSZ Z. RATAJCZAK

**Affiliations:** 1 Stem Cell Institute at the James Graham Brown Cancer Center, University of Louisville, Louisville, KY;; 2 Department of Pathology and Laboratory Medicine, University of Pennsylvania School of Medicine, Philadelphia, PA;; 3 Laboratory of Pathology, National Cancer Institute, Bethesda, MD, USA

**Keywords:** rhabdomyosarcoma, *DLK1-GTL2* locus, genomic imprinting

## Abstract

Parental imprinting of differentially methylated regions (DMRs) contributes to appropriate expression of several developmentally important genes from paternally or maternally derived chromosomes. Rhabdomyosarcoma (RMS) is the most common soft-tissue sarcoma in children and is associated with altered expression of certain parentally imprinted genes. As previously reported, RMS cells display loss of imprinting (LOI) of the DMR at the *IGF2-H19* locus, resulting in insulin-like growth factor 2 (*IGF2*) transcription from both paternally and maternally inherited chromosomes, and overall IGF2 overexpression. As the *DLK1-GTL2* locus is structurally similar to the *IGF2-H19* locus, the status of parental imprinting of the *DLK1-GTL2* locus was studied in RMS. We observed that while both embryonal and alveolar rhabdomyosarcomas (ERMS and ARMS, respectively) show LOI of the DMR at the *IGF2-H19* locus, imprinting of the DMR at the *DLK1-GTL2* locus varies in association with the histological subtype of RMS. We found that, while ERMS tumors consistently show LOI of the DMR at the *DLK1-GTL2* locus, ARMS tumors have erasure of imprinting (EOI) at this locus. These changes in imprinting status of the DLK1-GTL2 locus result in a higher *GTL2/DLK1* mRNA ratio in ARMS as compared to ERMS. This difference in imprinting elucidates a novel genetic difference between these two RMS subtypes and may provide a potential diagnostic tool to distinguish between these subtypes.

## Introduction

The delta-like homolog 1 (*DLK1*) and gene trap locus 2 (*GTL2*, also known as *MEG3*) gene cluster is located on human chromosome 14. The importance of the *DLK1-GTL2* locus in skeletal muscle development is supported by the fact that a single-nucleotide polymorphism at this locus deregulates its expression and leads to DLK1-mediated skeletal muscle hypertrophy, as seen in callipyge sheep (1).

Rhabdomyosarcoma (RMS), a family of cancers related to the skeletal muscle lineage, is the most common soft-tissue sarcoma in children. From a histological and clinical perspective, there are two major histological subtypes of RMS: embryonal rhabdomyosarcoma (ERMS) and alveolar rhabdomyosarcoma (ARMS) (2). Clinical evidence indicates that ARMS is more aggressive, shows more enhanced metastatic potential, and has a significantly worse outcome than ERMS. It has been postulated that there are different cellular origins for these tumors (3) but the molecular mechanisms responsible for the development of RMS are not well understood.

Evidence has accumulated that imprinted genes play a role in RMS pathogenesis (4,5). In the human genome, there are ∼80 genes that are imprinted and expressed only from the maternally or paternally derived chromosomes. This mechanism regulates the appropriate dosage and expression level of developmentally important genes in mammalian cells (6). The expression of imprinted genes is regulated by the imposition of epigenetic marks (DNA methylation) within differentially methylated regions (DMRs), which are regulatory CpG-rich regions in the gene locus (7–9). Most imprinted genes are methylated on maternally derived chromosomes and only a few (e.g., *IGF2* and *DLK1*) are methylated within DMRs on paternally derived chromosomes.

According to the parent-offspring conflict theory, while paternally expressed imprinted genes enhance embryo growth and size of the offspring, maternally expressed genes inhibit cell proliferation and negatively affect cell size (7–9). Based on this theory, during pregnancy, the paternal allele promotes increased body size, including muscle mass of the developing fetus, through expression of paternally imprinted genes. By contrast, the maternal allele conserves resources by epigenetic modulation of genes bearing maternal imprinting marks.

Comparison of the *IGF2-H19* and *DLK1-GTL2* tandem loci reveals significant similarities. The molecular structure of these imprinted loci is very similar. The DMRs for these tandem genes are both located between the two genes within each locus (between *IGF2* and *H19* and between *DLK1* and *GTL2*, respectively) (10,11), and these elements are thus called intergenic DMRs (IG-DMR). *IGF2* encodes insulin-like growth factor-2 (IGF-2) and *DLK1* encodes transmembrane delta-like 1 protein, which contains six epidermal growth factor (EGF) repeat motifs and is involved in cell differentiation in a juxtacrine/paracrine manner. Both *DLK1* and *IGF2* are transcribed from paternal chromosomes and are involved in skeletal muscle development as stimulators of skeletal muscle growth (12,13). By contrast, the two other genes in these tandem loci -H19 and GTL2- transcribe non-coding RNAs (ncRNAs) that are precursors of miRNAs that negatively regulate cell proliferation.

Loss of imprinting (LOI) of the IG-DMR at the *IGF2-H19* locus located at the 11p15.5 chromosome region is observed both in ERMS and ARMS. Furthermore, this LOI have been reported in both sporadic tumors and in RMS originating in Beckwith-Wiedemann syndrome patients (5). The result of this LOI is overexpression of IGF2, which acts as an autocrine growth factor in this tumor. Since the *IGF2-H19* locus is structurally similar to *DLK1-GTL2*, we investigated whether LOI of the DMR at the *DLK1-GTL2* locus also occurs in RMS. We observed that, while ERMS cells consistently showed LOI of the DMR at the *DLK1-GTL2* locus, ARMS cells displayed erasure of imprinting (EOI) in this regulatory region. Despite EOI of the IG-DMR within the *DLK1-GTL2* locus, ARMS cells highly express *DLK1* mRNA. Therefore, regulation of *DLK1* seems to be more complicated in RMS cells than initially thought. These findings have biological implications and may also have diagnostic utility.

## Materials and methods

### Patient samples

For 35 of the samples analyzed, frozen tumor samples were provided by the Pediatric Division of the Cooperative Human Tissue Network without patient identifiers. The only information provided was histologic diagnosis and fraction of tumor cells in a frozen section from the block from which the sample was taken. In the samples, the fraction of tumor cells varied from 65 to 90%. In 17 of the samples, the histologic subtype was embryonal rhabdomyosarcoma and in 18 of the samples the histologic subtype was alveolar rhabdomyosarcoma. In the latter 18 cases, the presence of the characteristic gene fusions was ascertained by RT-PCR revealing 14 cases with a PAX3-FOXO1 fusion and 4 cases with a PAX7-FOXO1 fusion (14).

### Combined bisulfite-restriction analysis (COBRA) and bisulfite-sequencing

DNA was isolated from frozen samples using the QIAamp system (Qiagen Inc., Valencia, CA). The DNA methylation status of IG-DMRs at the *DLK1-GLT2*, *IGF2-H19*, *LIT1*, *SNRPN* and *PEG1* loci were examined by COBRA analysis. In brief, 500 ng of genomic DNA was used for bisulfite modification using the EpiTect Bisulfite Kit (Qiagen Inc.) or the EZ DNA Methylation Kit (Zymo Research, Irvine, CA) according to the manufacturer’s instructions. IG-DMR regions were amplified by nested PCR (except for *LIT1*, for which only one round of PCR was performed) using bisulfite-treated gDNA as template and specific primers (Table I). For *DLK1-GLT2*, *IGF2-H19* and *PEG1*, both first-and second-round PCR were performed with 2 cycles of 2 min at 95°C, 1 min at 55°C and 1 min at 72°C; 35 cycles of 30 sec at 95°C, 1 min at 55°C and 1 min at 72°C; and 1 cycle of 10 min at 72°C. For *LIT1* and *SNRPC*, PCR was performed as previously described (16,17). The COBRA assay was performed by incubation of the final PCR products with BstUI restriction enzyme for 2 h and subsequent agarose gel electrophoresis to assess the cutting pattern.

For sequencing, amplicons from the above-described PCR reactions were eluted after agarose gel electrophoresis using the QIAquick Gel Extraction Kit (Qiagen Inc.). Eluted amplicons were ligated into the pCR^®^2.1-TOPO^®^ vector and transformed into TOP10 bacteria using a TOPO TA Cloning Kit (Invitrogen, Carlsbad, CA). The plasmids were prepared using a QIAprep Spin Miniprep Kit (Qiagen Inc.) and sequenced with M13 forward and reverse primers. The methylation pattern in DMRs was analyzed using CpG viewer software (18).

### Methylation-specific PCR of the IG-DMR at the DLK1-GTL2 locus

Methylation-specific PCR was performed on the bisulfite-treated genomic DNA with two different sets of primers recognizing either methylated or unmethylated alleles (Table I). PCR was performed at 2 cycles of 2 min at 95°C, 1 min at 55°C and 1 min at 72°C; 35 cycles of 30 sec at 95°C, 1 min at 55°C and 1 min at 72°C; and 1 cycle of 10 min at 72°C.

### Quantitative RT-PCR (qRT-PCR)

Total RNA from RMS patient samples was isolated using RNA STAT-60 (Tel-Test, Friendswood, TX) in accordance with manufacturer’s instructions. RNA was screened for expression of DLK1 and GTL2 using premade qRT-PCR assays from Applied Biosystems (Foster City, CA). All qRT-PCR assays were run on 384-well plates in the ABI Prism 7900 Sequence Detection System. The control 18S RNA was assayed in separate wells of the same run using a premade qRT-PCR assay (Applied Biosystems).

### Statistical analysis

Statistical analysis of the data was done using the Fisher’s exact test with p<0.05 as the criterion for significance.

## Results

### LOI of the DMR at the DLK1-GTL2 locus in ARMS and ERMS samples

**Figure 1.**
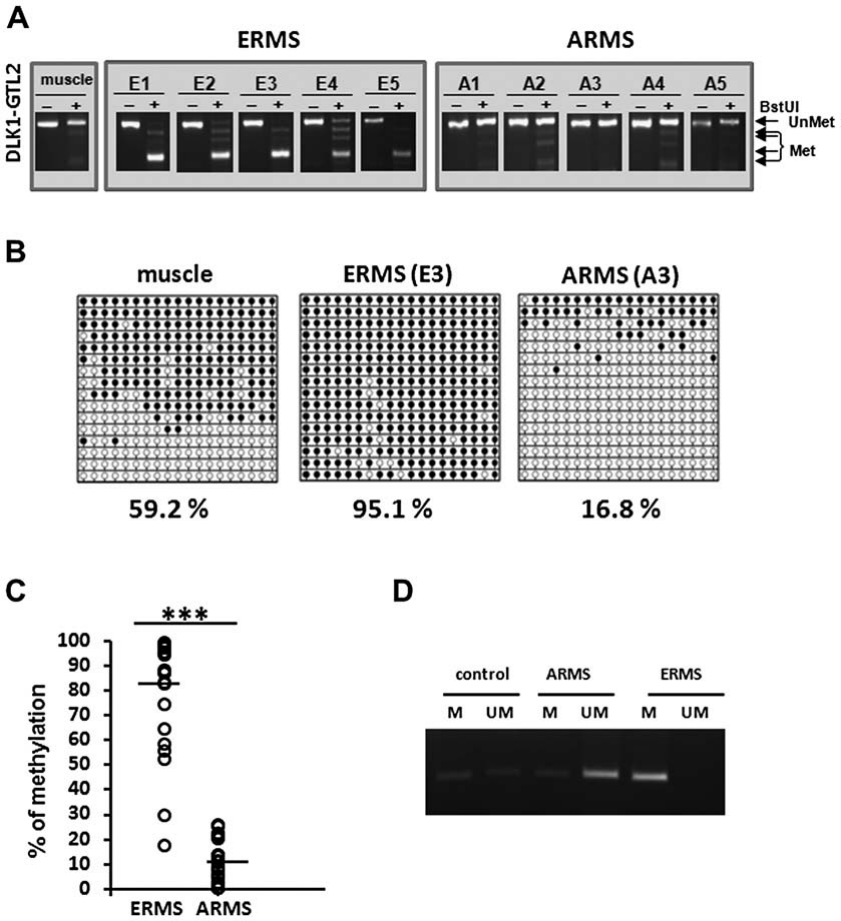
The methylation pattern of the IG-DMR at the *DLK1-GTL2* locus differs between ARMS and ERMS patient samples. (A) The COBRA assay was used to evaluate the methylation status of the IG-DMR at the *DLK1-GTL2* locus by employing the restriction enzyme BstUI. Methylated DNA (Met) is cleaved because methylcytosine is not deaminated to uracil by bisulfite treatment and thus the BstUI recognition site (CGCG) is maintained. By contrast, unmethylated DNA (UnMet) is not cleaved because the cytosine is deaminated to uracil and thus the sequence of the recognition site is changed. (B) An example of results from bisulfite modification of DNA followed by sequencing to evaluate DNA methylation of the IG-DMR at the *DLK1-GTL2* locus in human skeletal muscle samples and two RMS samples (ERMS and ARMS). Methylated and unmethylated CpG sites are shown as filled and open circles, respectively. Each horizontal line represents one subclone. The numbers under the bisulfite sequencing profiles indicate the percentage of methylated CpG sites. (C) Densitometric analysis of the methylation of the IG-DMR at the *DLK1-GTL2* locus based on the COBRA assay results for 22 ERMS and 23 ARMS samples. (D) Methylation-specific PCR analysis of the IG-DMR at the *DLK1-GTL2* locus in normal tissue (muscle) and two RMS samples (ERMS and ARMS). UM, unmethylated alleles; M, methylated alleles.

Since the *DLK1-GTL2* locus exhibits structural and molecular similarity to the *IGF2-H19* locus, we investigated the imprinting status of the IG-DMR at the *DLK1-GTL2* locus, which is located between the two genes and controls coordinated expression of both genes at this locus (19). To address this question, we performed the COBRA assay, in which PCR-amplified bisulfite-treated DNA is cut with an appropriate restriction enzyme (*Bst*UI) to determine whether the fragment has retained or lost the corresponding CpG-containing restriction enzyme recognition site. The digested DNA was subsequently separated and visualized by agarose gel electrophoresis. As shown in Fig. 1A, the *Bst*U1 site was cut in all five ERMS samples and thus this region was hypermethylated in these cases. As there is often no evidence of a residual uncut of hypomethylated allele, this finding is indicative of LOI in the ERMS cases. By contrast, all five analyzed ARMS patient samples have lost the BstU1 recognition site and thus exhibited hypomethylation of the IG-DMR at the *DLK1-GTL2* locus. On the other hand since there is often no evidence of a residual cut of hypermethylated allele, this finding is consistent with elimination of imprinting (EOI) in these ARMS cases.

To further confirm these imprinting changes at the *DLK1-GTL2* locus in RMS, we employed bisulfite modification of DNA followed by sequencing of selected ERMS (patient E3) and ARMS (patient A3) samples. The PCR-amplified bisulfite-treated DNA fragments were subcloned and multiple subclones were individually sequenced. These sequencing results further supported our COBRA results, as all the copies of the IG-DMR at the *DLK1-GTL2* locus were hypermethylated in the ERMS case (consistent with LOI), and were hypomethylated in the ARMS case (consistent with EOI) (Fig. 1B). As expected, DNA methylation of IG-DMRs in human skeletal muscle was ∼60% (19).

After these initial studies, to further confirm our observation we analyzed an additional 17 ERMS and 18 ARMS patient samples by the COBRA assay. Combined densitometric analysis of all analyzed patient samples showed a highly relevant difference in methylation pattern between ARMS and ERMS. The average methylation of ARMS samples was ∼10%, whereas for ERMS samples, we observed 80% methylation (Fig. 1C). Therefore, these results demonstrate that the methylation pattern of the IG-DMR significantly differs between the two subtypes of RMS.

Since distinguishing between ARMS and ERMS currently requires histopathology analysis of patient samples, we developed methylation-specific PCR primers that enable quick and specific analysis of the methylation status of the *DLK1-GTL2* locus and thus can be potentially employed in RMS diagnostics (Fig. 1D).

### The methylation pattern of the DMRs at the IGF2-H19, PEG1, LIT1 and SNRPN loci do not show differences between ARMS and ERMS samples

**Figure 2.**
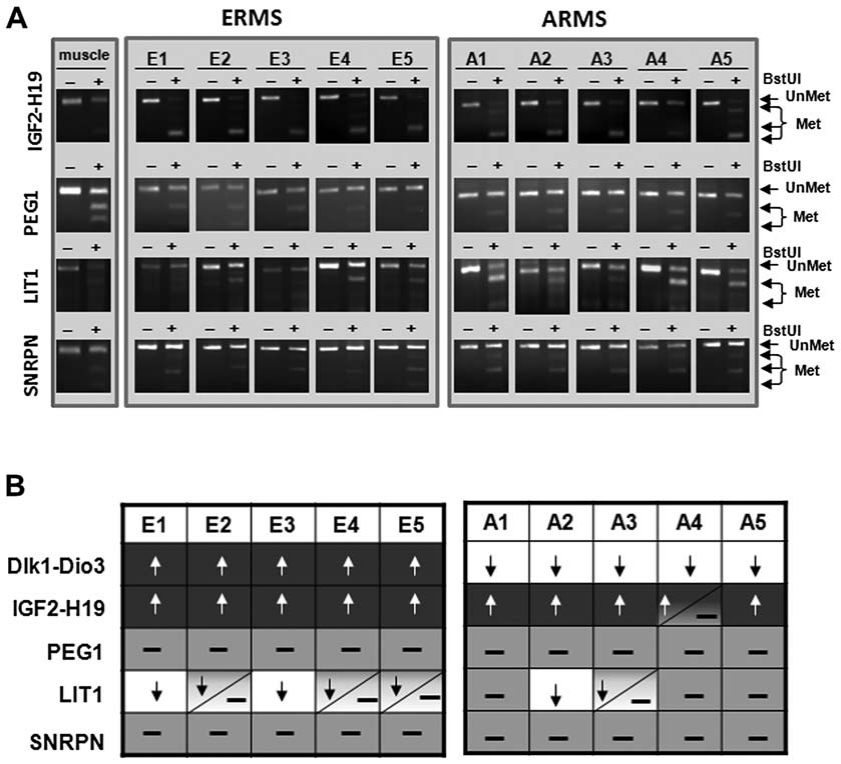
Methylation pattern of the DMRs at the *IGF2-H19*, *PEG1*, *LIT1* and *SNRPN* loci in ERMS and ARMS patient samples. (A) COBRA assay for DMRs at the *IGF2-H19*, *PEG1*, *LIT1* and *SNRPN* loci in the presence of BstUI restriction enzyme. The unmethylated DNA (UnMet) was not cleaved, in contrast to methylated DNA (Met), because of a sequence change in the site recognized by the restriction enzyme after the bisulfite reaction. (B) Summary of methylation patterns of DMRs at the *DLK1-GTL2*, *IGF2-H19*, *PEG1*, *LIT1* and *SNRPN* loci in DNA samples from normal skeletal muscles and patient DNA samples (ERMS and ARMS). White color with black arrow indicates hypomethylation (EOI), light grey indicates normal status (50%) and dark color with white arrow indicates hypermethylation (LOI).

Next, by applying additional COBRA assays on the same DNA samples derived from ERMS and ARMS tumors, we performed an analysis of the methylation status of the DMRs at other imprinted loci, such as *IGF2-H19*, *PEG1*, *LIT1* and *SNRPN*. As expected, we confirmed LOI of the DMR within *IGF2-H19* in all of the samples and at the same time some degree of hypomethylation LOI of the *LIT1* DMR in most of the samples. At the same time, we did not observe any significant changes in the methylation state of *PEG1* and *SNRPN* loci (Fig. 2A).

Fig. 2B summarizes the methylation pattern of DMRs at all loci tested by us in ERMS and ARMS tumor samples and demonstrates that, for all of the DMRs evaluated, imprinting at the *DLK1-GTL2* locus differs between ERMS and ARMS and may be of some diagnostic importance for example as an addition to fusion assay.

### LOI of the DMR at the DLK1-GTL2 locus correlates with changes in GTL2/DLK1 mRNA ratio

**Figure 3.**
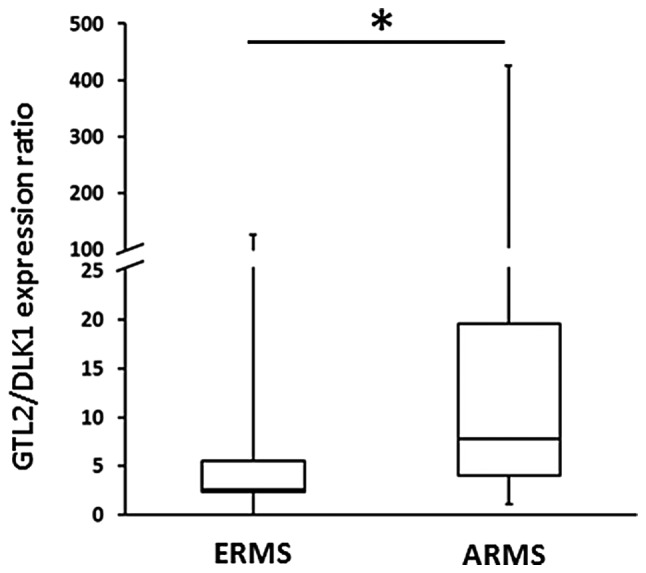
qRT-PCR analysis of expression of DLK1-GTL2 genes regulated by imprinting. GTL2/DLK1 mRNA ratio in ARMS and ERMS patient samples; *p<0.05.

Finally, to see whether changes in imprinting of particular DMRs are translated into changes in RNA transcription, we performed qRT-PCR analysis of the most important imprinted genes in RNA extracts isolated from ERMS and ARMS samples (Fig. 3). To address this question we compared the GTL2/DLK1 ratio in a large panel of ERMS and ARMS tumors. The Fisher’s exact test analysis revealed a higher GTL2/DLK1 ratio in ARMS patients as compared to ERMS; this finding is consistent with LOI at the DLK1-GTL2 locus.

## Discussion

The tandem *DLK1-GTL2* gene locus is part of a large genomic region located on chromosome 14 (14q32) known as *DLK1-DIO3* (20), which contains three paternally imprinted and expressed genes, *DLK1*, *RTL1* and *DIO3*, as well as three maternally expressed genes, *GTL2* (*MEG3*), *MEG8* (*RIAN*), and the antisense gene *RTL1*. Moreover, in addition to two long non-coding intergenic RNAs (*GTL2* and *MEG8*), this region contains one of the largest miRNA clusters in the human genome, encoding a total of 54 miRNAs, which play an important role in tissue homeostasis and, when aberrantly expressed, are involved in tumorigenesis (21). We focused on the *DLK1-GTL2* locus in the 3’ end of this region, which is regulated by imprinting of the IG-DMR. The proper expression of genes in this locus from the paternally and maternally inherited genes *DLK1* and *GTL2*, respectively, is crucial for normal development (19,22) and pluripotency of stem cells (23).

Disordered imprinting has been implicated in the pathogenesis of pediatric cancers. In particular, epigenetic alterations in imprinting within the *IGF2-H19* locus have been observed in pediatric cancers, such as RMS and nephroblastoma (Wilm’s tumor), which develop spontaneously or as a part of Beckwith-Wiedemann syndrome (4). Based on these observations, we became interested in potential alterations to imprinting at the *DLK1-GTL2* locus, which is structurally similar to the *IGF2-H19* locus. Such alterations of imprinting are important because LOI of the IG-DMR at the *DLK1-GTL2* locus may result in overexpression of *DLK1*, which stimulates development of skeletal muscle tissue (12), and downregulation of *GTL2*, which, as recently postulated, may have an anti-oncogenic function (21,24). In the case of EOI of the IG-DMR at the *DLK1-GTL2* locus, we would expect to see a reversed pattern of expression for these genes, downregulation of *DLK1* and upregulation of *GTL2*.

In our studies, performed on DNA samples from 23 ERMS and 23 ARMS tumors, we observed that the IG-DMR at the *DLK1-GTL2* locus is hypermethylated (LOI) in ERMS and hypomethylated (EOI) in ARMS cells. Therefore, in contrast to the LOI that is consistently observed within the *IGF2-H19* locus in both ERMS and ARMS samples, methylation of the IG-DMR at the *DLK1-GTL2* locus is RMS-subtype specific. This different pattern of methylation could have some diagnostic value. By employing a specific pair of primers that recognize methylated DNA sequences, we have demonstrated that it is possible to quickly phenotype both subtypes of RMS by PCR.

To address if these changes in imprinting at *DLK1-GTL2* locus correspond to changes in mRNA expression we analyzed by employing RQ-PCR the GTL2/DLK1 ratio in 10 fusion-negative and 16 PAX3-FOXO1 fusion-positive ARMS samples. Our mRNA expression data correlated with imprinting status of the *DLK1-GTL2* locus and demonstrated a significantly higher GTL2/DLK1 ratio in ARMS tumor samples compared to ERMS tumors.

In conclusion our data indicate that DLK1/GTL2 locus is differently regulated by imprinting in ARMS vs. ERMS cells. We developed an easy PCR-based assay to phenotype ERMS and ARMS, based on the methylation status of the IG-DMR at the *DLK1-GTL2* locus. However, we are aware that in addition to epigenetic regulation some other mechanisms could be involved. In support of this, an insulator protein located 18 kb upstream of the *DLK1-GTL2* locus, which plays an important role in regulating imprinting of this locus, has been identified in human acute leukemia cells (25). Thus, regulation of expression of imprinted genes in RMS as well as (probably) in other tumor cells requires further study.

## Figures and Tables

**Table I.** Sequences of primers employed for bisulfite sequencing and methylation-specific PCR.

Region		Sequence	(Refs.)
Bisulfite sequencing			
*DLK1-GTL2*	Outer	F: TGG GAA TTG GGG TAT TGT TTA T R: AAA CAA TTT AAC AAC AAC TTT CCT C	(19)
	Inner	F: GTT AAG AGT TTG TGG ATT TGT GAG AAA TG R: CTA AAA ATC ACC AAA ACC CAT AAA ATC AC	✓
*IGF2-H19*	Outer	F: AGG TGT TTT AGT TTT ATG GAT GAT GG R: TCC TAT AAA TAT CCT ATT CCC AAA TAA CC	(15)
	Inner	F: TGT ATA GTA TAT GGG TAT TTT TGG AGG TTT R: H19-OR was used	(15)
*LIT1*	(One PCR)	F: GTG TTA IGG IGG TGG AGA TTT TGT R: AAC TIA AAACACIAACCAATTCTCTA	(16)
*PEG1*	Outer	F: TTG TTG TTG GTT AGT TTT GTA TGG TT R: AAA AAT AAC ACC CCC TCC TCA AAT	(15)
	Inner	F: PEG1-OF was used R: CCC AAA AAC AAC CCC AAC TC	(15)
*SNRPN*	Outer	F: GTG TTG TGG GGT TTT AGG GGT TTA G R: CTC CCC AAA CTA TCT CTT AAA AAA AAC C	(17)
	Inner	F: AGG GAG TTG GGA TTT TTG TAT TG R: SNRPN-OR was used	(17)
Methylation-specific PCR			
*DLK1-GTL2*	Methylated	F: TAT TTT AAG ATT GTT AGT TTT TTC GC R: AAA ACC CAA CCC AAT AAA CG	✓
	Unmethylated	F: GTTT TA TTT TAA GAT TGT TAG TTT TTT R: CAA AAC CCA ACC CAA TAA ACA	✓

✓, Primers designed in this study; Outer, outer primers; Inner, inner primers; F, forward; R, reverse.

## References

[1] Davis E, Jensen CH, Schroder HD (2004). Ectopic expression of DLK1 protein in skeletal muscle of padumnal heterozygotes causes the callipyge phenotype. Curr Biol.

[2] Davicioni E, Anderson MJ, Finckenstein FG (2009). Molecular classification of rhabdomyosarcoma - genotypic and phenotypic determinants of diagnosis: a report from the Children’s Oncology Group. Am J Pathol.

[3] Hettmer S, Wagers AJ (2010). Muscling in: Uncovering the origins of rhabdomyosarcoma. Nat Med.

[4] Chung WY, Yuan L, Feng L, Hensle T, Tycko B (1996). Chromosome 11p15.5 regional imprinting: comparative analysis of KIP2 and H19 in human tissues and Wilms’ tumors. Hum Mol Genet.

[5] Casola S, Pedone PV, Cavazzana AO (1997). Expression and parental imprinting of the H19 gene in human rhabdomyosarcoma. Oncogene.

[6] Ishida M, Moore GE (2013). The role of imprinted genes in humans. Mol Aspects Med.

[7] Reik W, Walter J (2001). Genomic imprinting: parental influence on the genome. Nat Rev Genet.

[8] Pannetier M, Feil R (2007). Epigenetic stability of embryonic stem cells and developmental potential. Trends Biotechnol.

[9] Delaval K, Feil R (2004). Epigenetic regulation of mammalian genomic imprinting. Curr Opin Genet Dev.

[10] Geuns E, De Temmerman N, Hilven P, Van Steirteghem A, Liebaers I, De Rycke M (2007). Methylation analysis of the intergenic differentially methylated region of DLK1-GTL2 in human. Eur J Hum Genet.

[11] Sasaki H, Ishihara K, Kato R (2000). Mechanisms of Igf2/H19 imprinting: DNA methylation, chromatin and long-distance gene regulation. J Biochem.

[12] Waddell JN, Zhang P, Wen Y (2010). Dlk1 is necessary for proper skeletal muscle development and regeneration. PLoS One.

[13] Markljung E, Jiang L, Jaffe JD (2009). ZBED6, a novel transcription factor derived from a domesticated DNA transposon regulates IGF2 expression and muscle growth. PLoS Biol.

[14] Duan F, Smith LM, Gustafson DM (2012). Genomic and clinical analysis of fusion gene amplification in rhabdomyosarcoma: a report from the Children’s Oncology Group. Genes Chromosomes Cancer.

[15] Kerjean A, Dupont JM, Vasseur C (2000). Establishment of the paternal methylation imprint of the human H19 and MEST/PEG1 genes during spermatogenesis. Hum Mol Genet.

[16] Higashimoto K, Urano T, Sugiura K (2003). Loss of CpG methylation is strongly correlated with loss of histone H3 lysine 9 methylation at DMR-LIT1 in patients with Beckwith-Wiedemann syndrome. Am J Hum Genet.

[17] Kobayashi H, Sato A, Otsu E (2007). Aberrant DNA methylation of imprinted loci in sperm from oligospermic patients. Hum Mol Genet.

[18] Carr IM, Valleley EM, Cordery SF, Markham AF, Bonthron DT (2007). Sequence analysis and editing for bisulphite genomic sequencing projects. Nucleic Acids Res.

[19] Kagami M, Sekita Y, Nishimura G (2008). Deletions and epimutations affecting the human 14q32.2 imprinted region in individuals with paternal and maternal upd(14)-like phenotypes. Nat Genet.

[20] Ogata T, Kagami M, Ferguson-Smith AC (2008). Molecular mechanisms regulating phenotypic outcome in paternal and maternal uniparental disomy for chromosome 14. Epigenetics.

[21] Benetatos L, Hatzimichael E, Londin E (2013). The microRNAs within the DLK1-DIO3 genomic region: involvement in disease pathogenesis. Cell Mol Life Sci.

[22] Li L, Forman SJ, Bhatia R (2005). Expression of DLK1 in hematopoietic cells results in inhibition of differentiation and proliferation. Oncogene.

[23] Liu L, Luo GZ, Yang W (2010). Activation of the imprinted Dlk1-Dio3 region correlates with pluripotency levels of mouse stem cells. J Biol Chem.

[24] Zhou Y, Zhang X, Klibanski A (2012). MEG3 noncoding RNA: a tumor suppressor. J Mol Endocrinol.

[25] Khoury H, Suarez-Saiz F, Wu S, Minden MD (2010). An upstream insulator regulates DLK1 imprinting in AML. Blood.

